# Cyclic voltammograms and electrochemical data of Fe^II^ polypyridine complexes

**DOI:** 10.1016/j.dib.2020.105754

**Published:** 2020-05-21

**Authors:** Jeanet Conradie, Karel G. von Eschwege

**Affiliations:** Department of Chemistry, PO Box 339, University of the Free State, Bloemfontein, 9300, South Africa

**Keywords:** Iron bipyridyl, Iron phenanthroline, Photocatalyst, Redox potential prediction, Redox indicator

## Abstract

Data in this article is associated with our research article, Electronic Properties of Fe Charge Transfer Complexes – a Combined Experimental and Theoretical Approach [1].

The oxidation and reduction potentials of fourteen Fe^II^ complexes are presented here, as extracted from the redox data obtained from its associated cyclic voltammograms, which were measured at scan rates varying from 0.05 V.s^−1^ to 5.00 V.s^−1^, under similar experimental conditions. Acetonitrile was used as solvent, and tetrabutylammonium hexafluorophosphate as supporting electrolyte. All data are reported *versus* the Fe^II^ redox couple in ferrocene.

**Specifications Table**

**Table:** 

**Subject**	Chemistry
**Specific subject area**	Electrochemistry
**Type of data**	Table
	Graph
	Figure
**How data were acquired**	BAS 100B/W electrochemical analyzer
**Data format**	Raw
	Analyzed
**Parameters for data collection**	All samples were synthesized.
	The acetonitrile solvent-electrolyte solutions in the electrochemical cell were degassed with Argon gas, for approximately 10 minutes. After addition of sample, degassing were continued for yet an additional ca 3 minutes.
	The cell was kept under a blanket of Argon gas for the duration of all electrochemical analysis.
**Description of data collection**	A 2 mL electrochemical cell was used for all electrochemical analyses of the samples, which consisted of a glassy carbon working electrode, Pt reference electrode and a Pt auxiliary electrode.
	The electrochemical cell was controlled by a BAS 100 B/W electrochemical analyzer. Data was processed in Excel, for purposes of data analysis and diagram presentation.
**Data source location**	Department of Chemistry
	University of the Free State
	Bloemfontein
	South Africa
**Data accessibility**	With the article
**Related research article**	H. Ferreira, K.G. von Eschwege, J. Conradie
	Electronic Properties of Fe Charge Transfer Complexes
	– a Combined Experimental and Theoretical Approach
	DOI: 10.1016/j.electacta.2016.09.034

**Value of the Data**

• Cyclic voltammograms provide oxidation potential data of the Fe^II/III^ redox couple of fourteen polypyridine Fe^II^ complexes at scan rates 0.05 – 5.0 Vs^−1^.
• The ease of oxidation of Fe^II^ in the fourteen Fe^II^ polypyridine complexes are determined by the functional groups on the polypyridine ligands.
• The suitability of the complex as a catalyst is determined by the redox data of the complexes. As opposed to the platinum group metals, iron is an earth-abundant and eco-friendly metal.
• The suitability of the complex as redox indicator is determined by the redox data of the complexes.
• Oxidation potential data, amongst others, of metal-to-ligand charge transfer complexes determine if complexes qualify for application as photo-active mediators or dyes in dye sensitized solar cells.
• Metal-to-ligand charge transfer complexes also serve as important catalysts in photo-catalytic reduction of CO_2_ and/or H_2_O in the production of environmentally friendly fuels.

## 1 Data Description

**Fig. 1:**
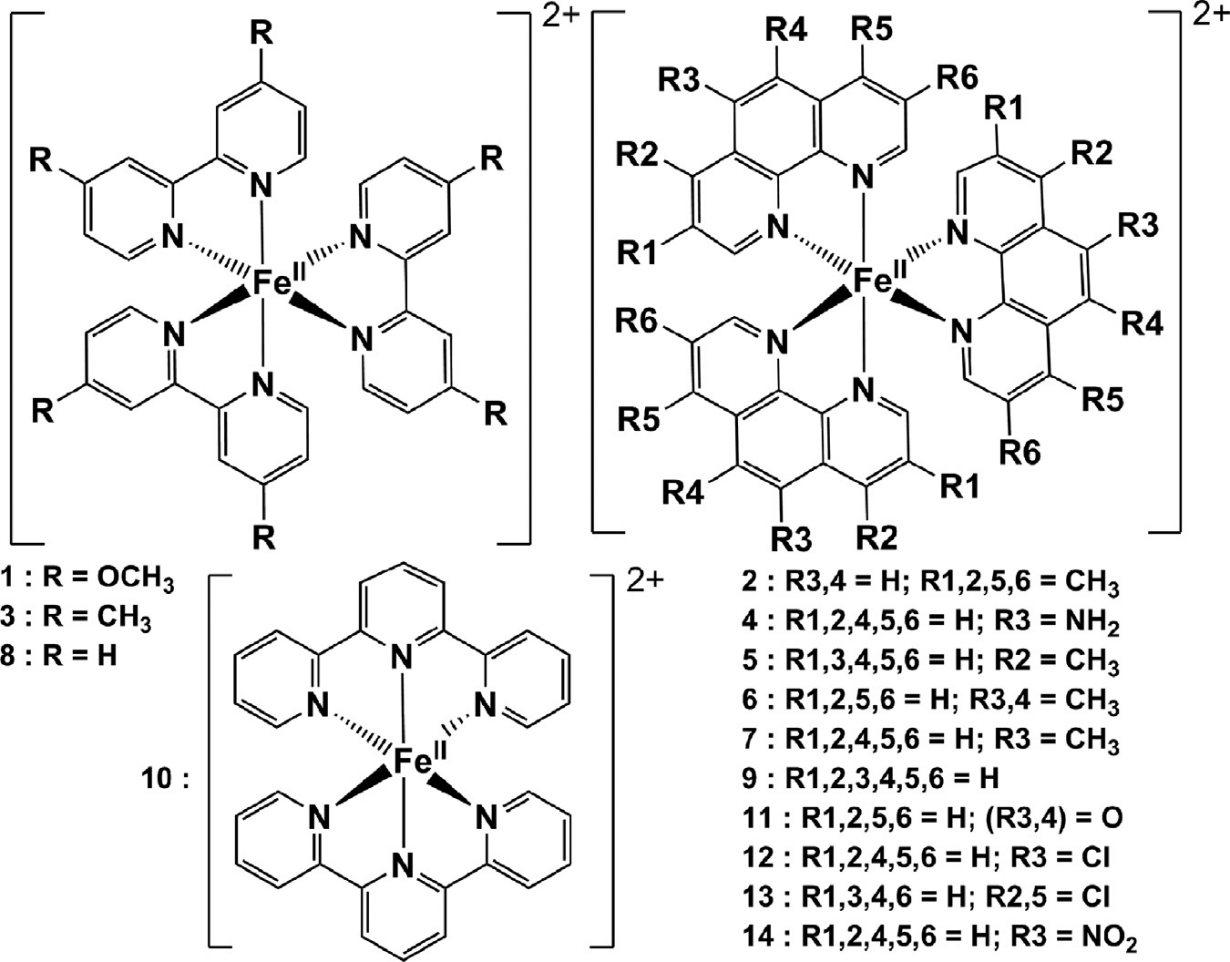
Complex numbering and structure of Fe^II^ polypyridine complexes.

**Fig. 2:**
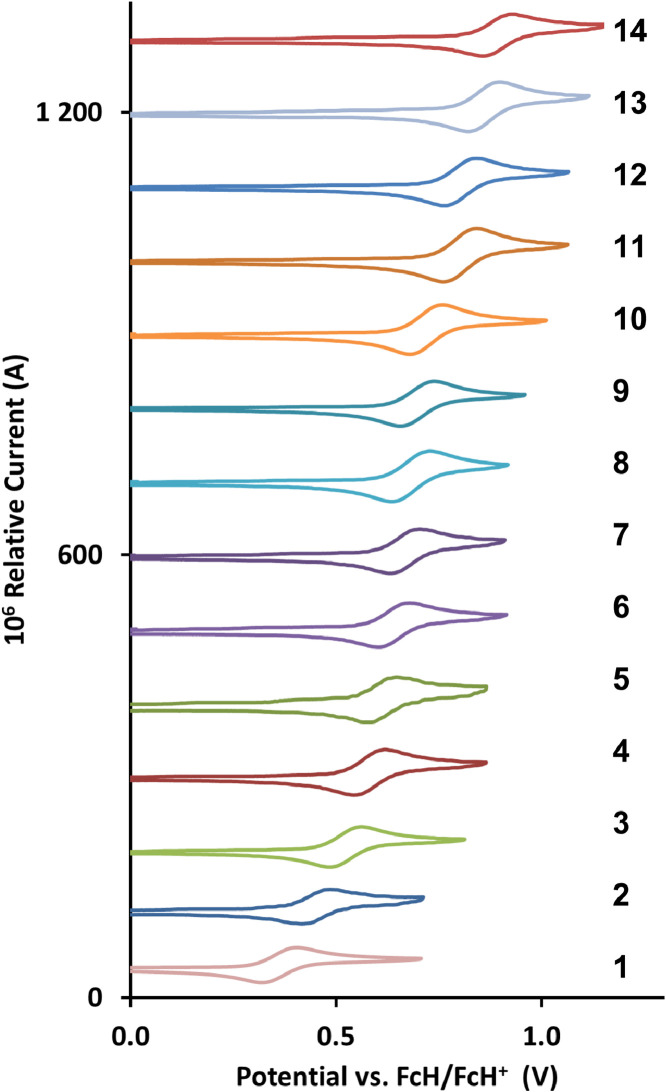
Stacked *c*yclic voltammograms of Fe^II^ polypyridyl complexes 1 - 14 in CH_3_CN as solvent, with [NBu_4_][PF_6_]) as supporting electrolyte, at a scan rate of 0.1 V/s initiated in the positive direction.

**Fig. 3:**
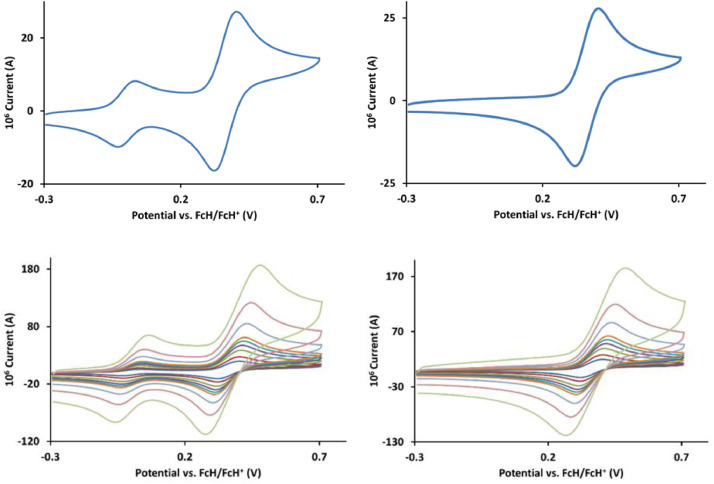
Cyclic voltammograms of *tris*(4,4’-dimethoxy-2,2’-bipyridine)iron(II) perchlorate, [Fe(4,4’-OMe-phen)_3_](ClO_4_)_2_, 1, in CH_3_CN as solvent with [NBu_4_][PF_6_]) as supporting electrolyte. *Top left*: 0.1 V/s scan rate with ferrocene as internal standard. *Top right*: 0.1 V/s scan rate without ferrocene as internal standard. *Bottom left*: scan rates 0.05, 0.10, 0.20, 0.30, 0.40, 0.50, 1.00, 2.00 and 5.00 V/s with ferrocene as internal standard. *Bottom right*: scan rates 0.05, 0.10, 0.20, 0.30, 0.40, 0.50, 1.00, 2.00 and 5.00 V/s without ferrocene as internal standard. All scans initiated in the positive direction from -0.3 V.

**Fig. 4:**
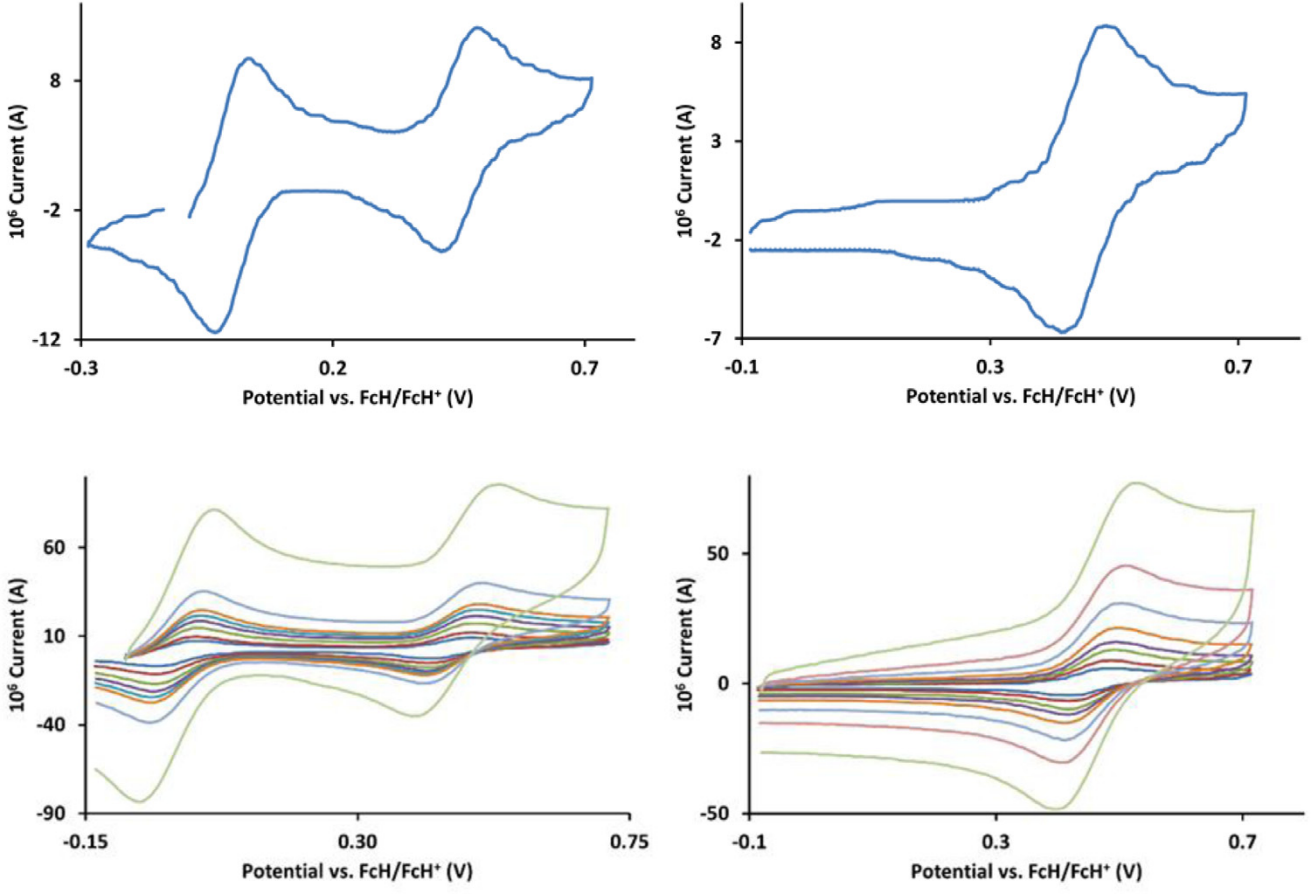
Cyclic voltammograms of *tris*(3,4,7,8-tetramethyl-1,10-phenanthroline)iron(II) perchlorate, [Fe(3,4,7,8-Me-phen)_3_](ClO_4_)_2_, 2, in CH_3_CN as solvent with [NBu_4_][PF_6_]) as supporting electrolyte. Details are provided in the caption of Fig. 3.

**Fig. 5:**
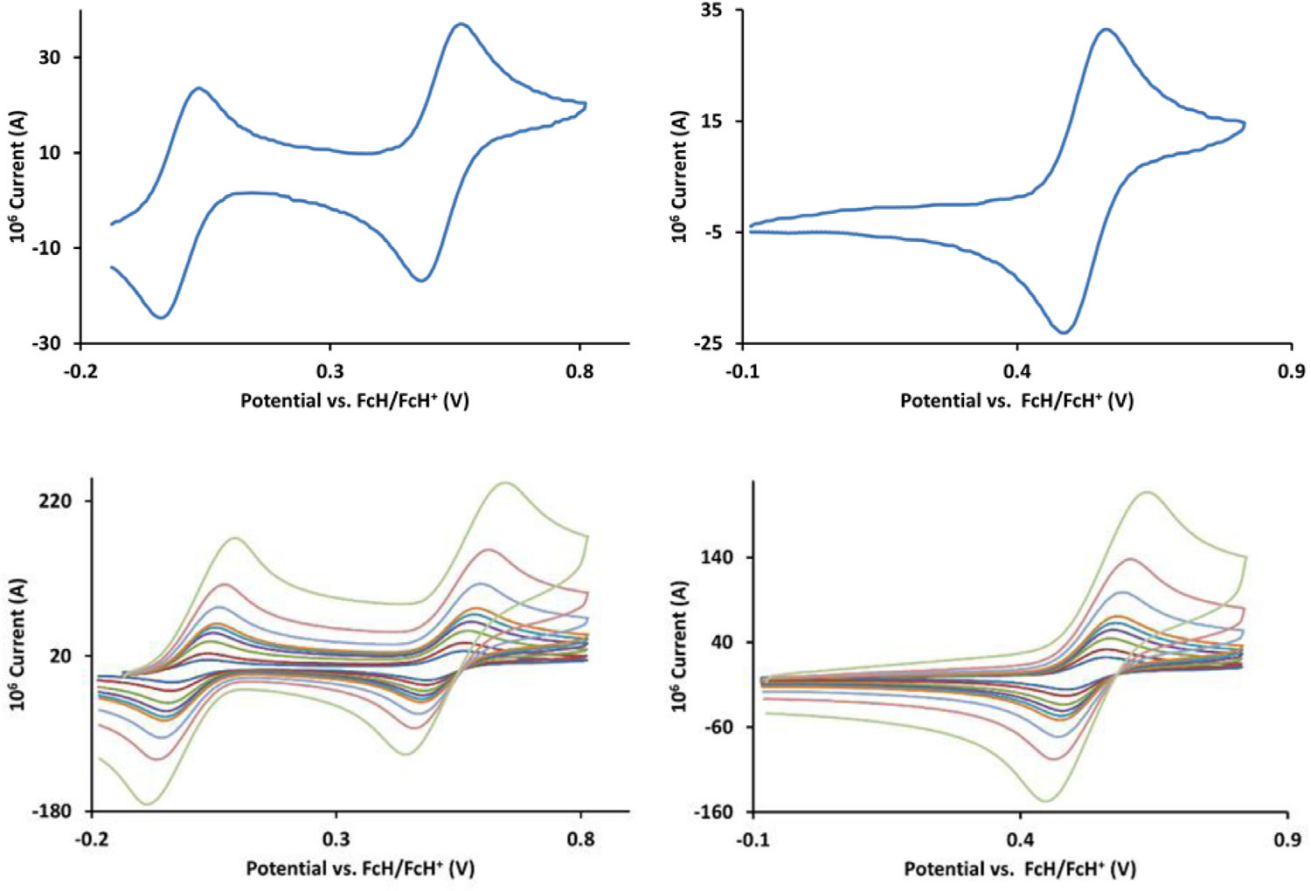
Cyclic voltammograms of *tris*(4,4’-dimethyl-2,2’-bipyridine)iron(II) perchlorate, [Fe(4,4’-Me-phen)_3_](ClO_4_)_2_, 3, in CH_3_CN as solvent with [NBu_4_][PF_6_]) as supporting electrolyte. Details are provided in the caption of Fig. 3.

**Fig. 6:**
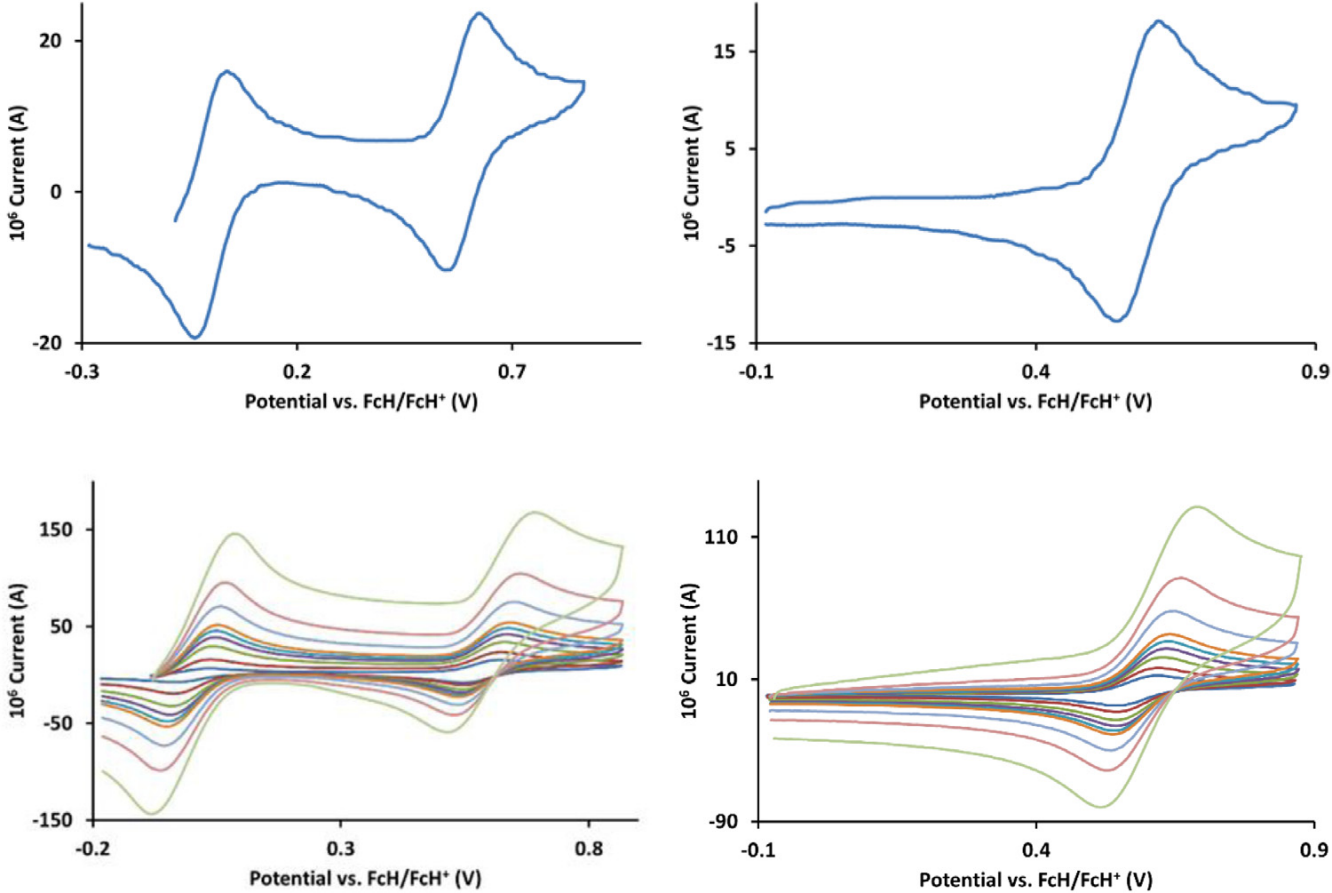
Cyclic voltammograms of *tris*(5-amine-1,10-phenanthroline)iron(II) perchlorate, [Fe(5-NH_2_-phen)_3_](ClO_4_)_2_, 4, in CH_3_CN as solvent with [NBu_4_][PF_6_]) as supporting electrolyte. Details are provided in the caption of Fig. 3.

**Fig. 7:**
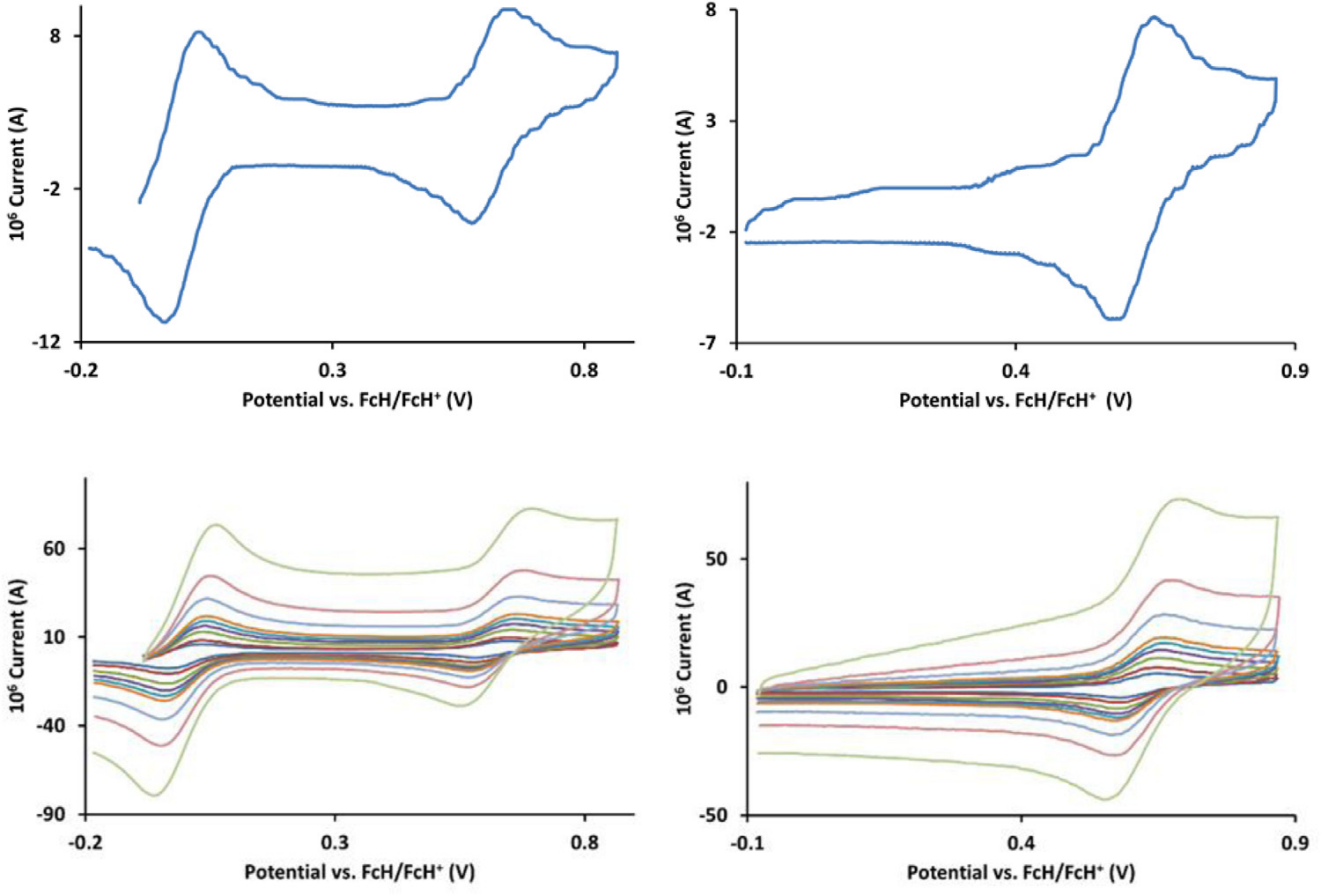
Cyclic voltammograms of *tris*(4-methyl-1,10-phenanthroline)iron(II) perchlorate, [Fe(4-Me-phen)_3_](ClO_4_)_2_ 5, in CH_3_CN as solvent with [NBu_4_][PF_6_]) as supporting electrolyte. Details are provided in the caption of Fig. 3.

**Fig. 8:**
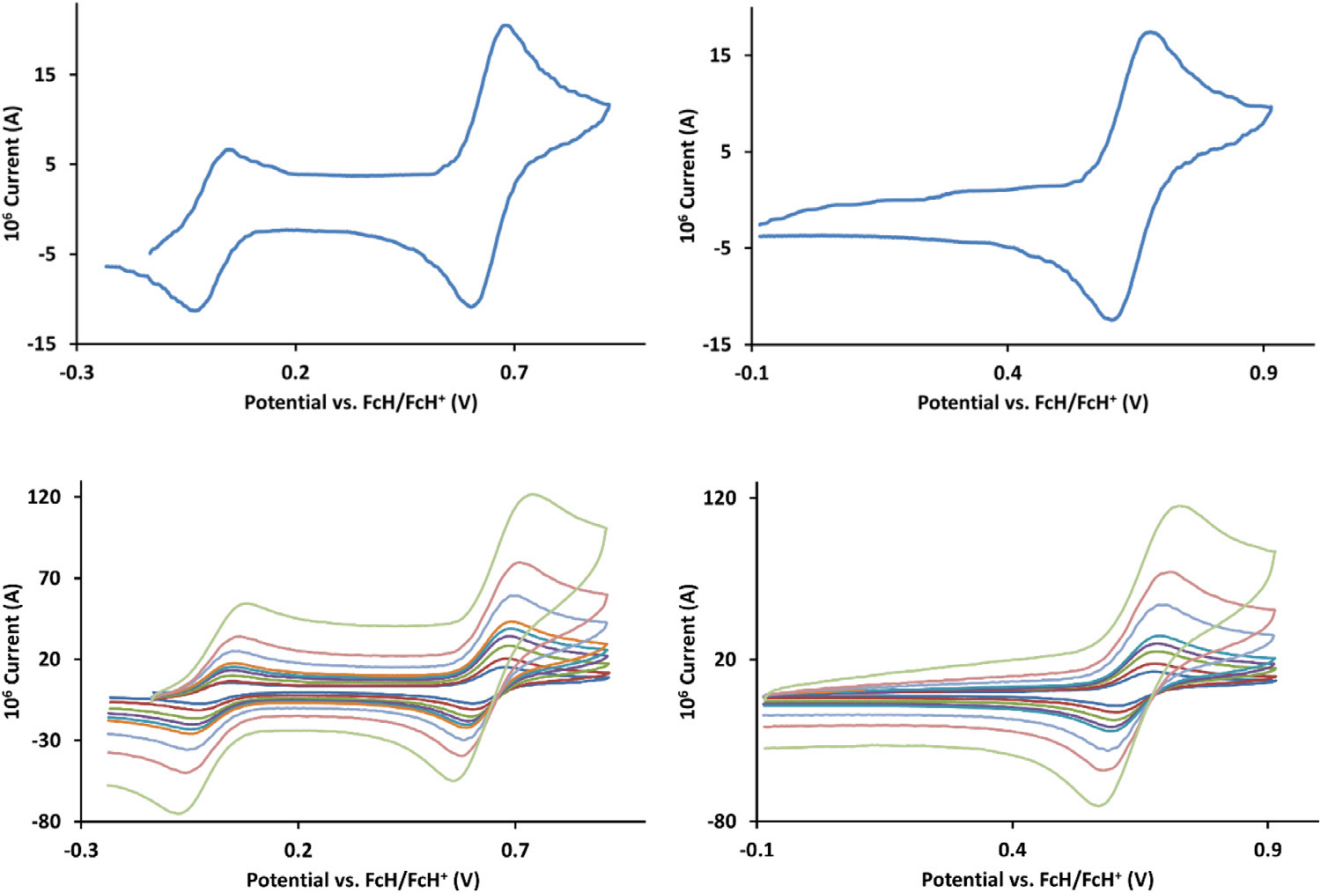
Cyclic voltammograms of *tris*(5,6-dimethyl-1,10-phenanthroline)iron(II) perchlorate, [Fe(5,6-Me-phen)_3_](ClO_4_)_2_, 6, in CH_3_CN as solvent with [NBu_4_][PF_6_]) as supporting electrolyte. Details are provided in the caption of Fig. 3.

**Fig. 9:**
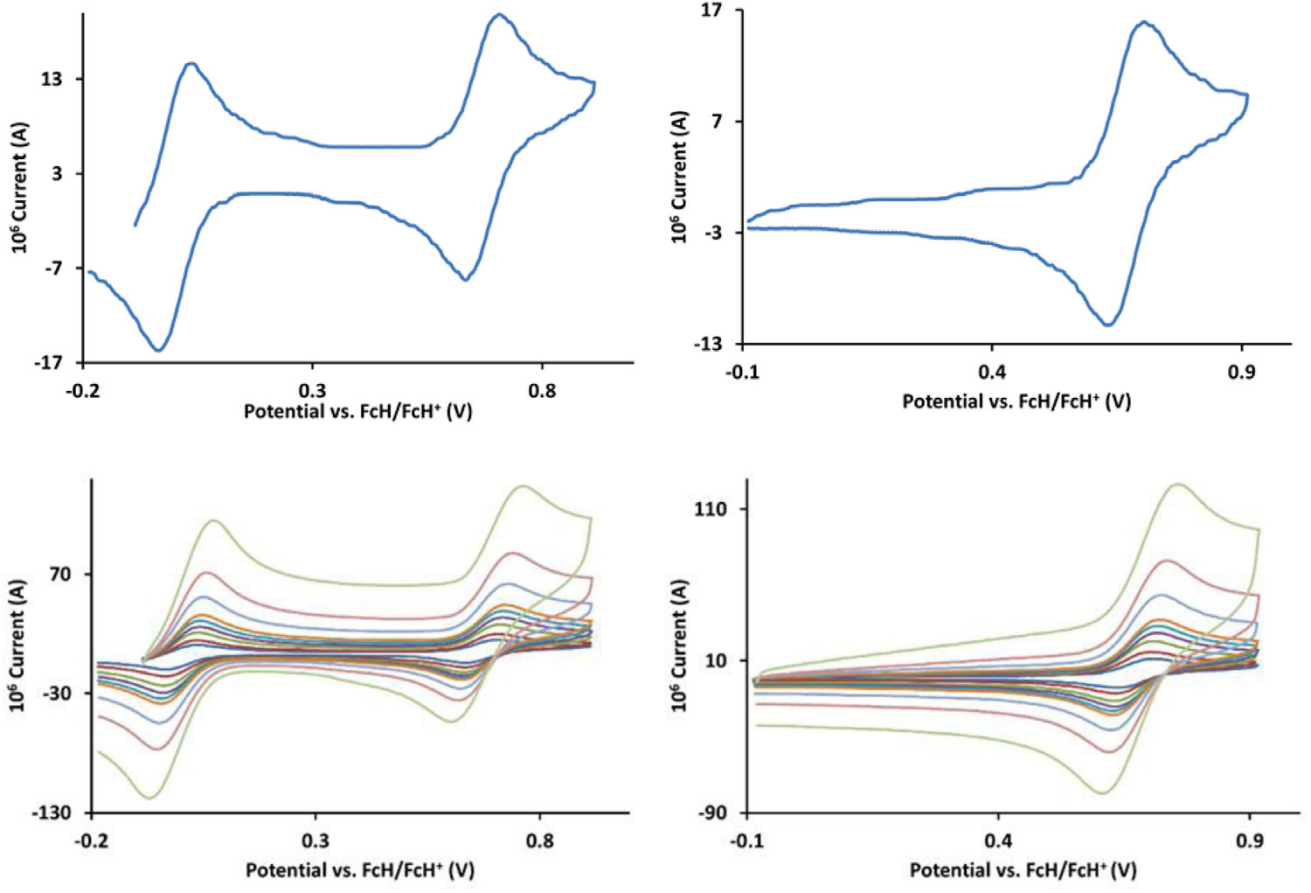
Cyclic voltammograms of *tris*(5-methyl-1,10-phenanthroline)iron(II) perchlorate, [Fe(5-Me-phen)_3_](ClO_4_)_2_, 7, in CH_3_CN as solvent with [NBu_4_][PF_6_]) as supporting electrolyte. Details are provided in the caption of Fig. 3.

**Fig. 10:**
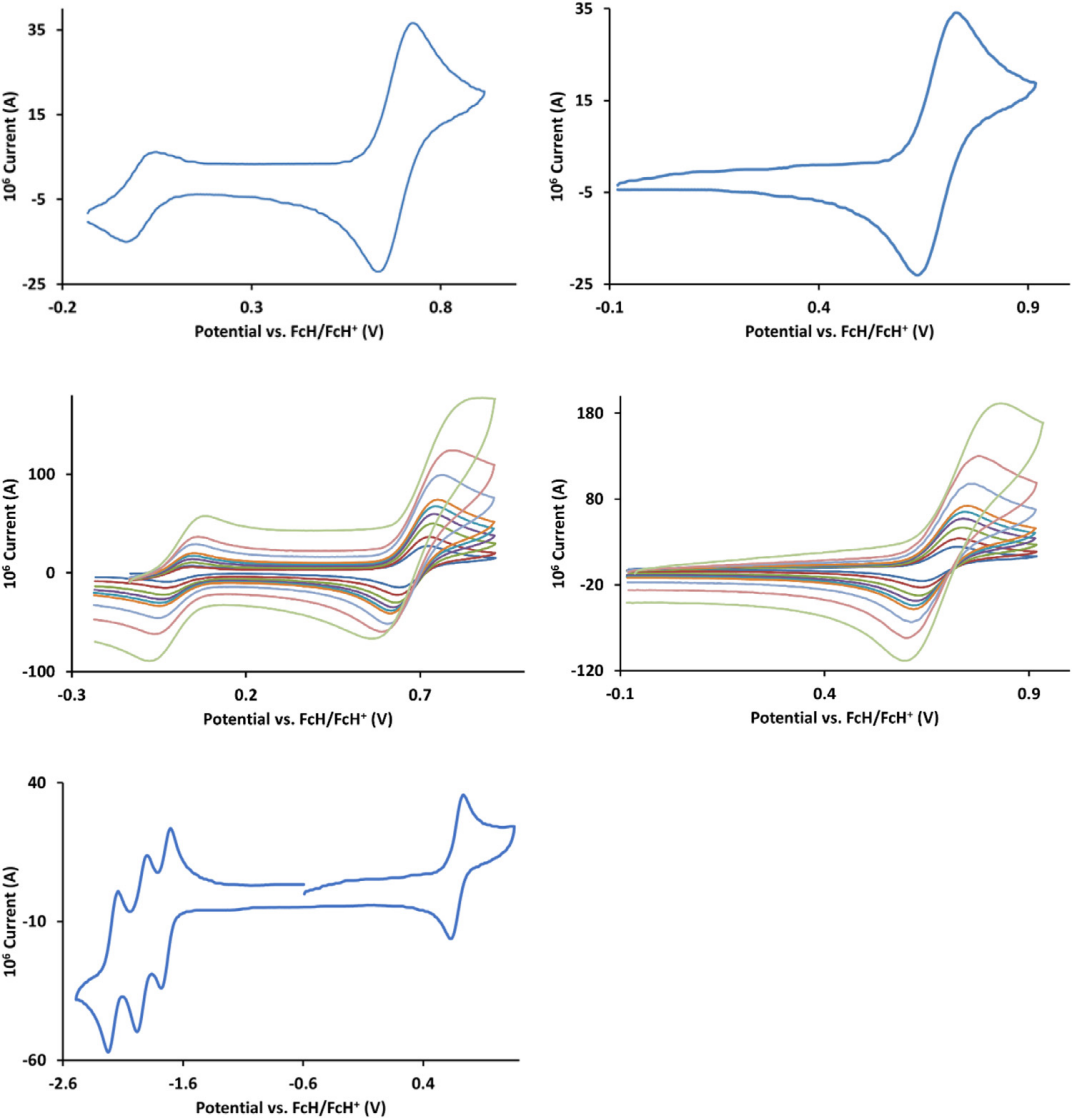
Cyclic voltammograms of *tris*(2,2’-bipyridine)iron(II) perchlorate, [Fe(bipy)_3_](ClO_4_)_2_, 8, in CH_3_CN as solvent with [NBu_4_][PF_6_]) as supporting electrolyte. *Top left*: 0.1 V/s scan rate with ferrocene as internal standard. *Top right*: 0.1 V/s scan rate without ferrocene as internal standard. *Middle left*: scan rates 0.05, 0.10, 0.20, 0.30, 0.40, 0.50, 1.00, 2.00 and 5.00 V/s with ferrocene as internal standard. *Middle right*: scan rates 0.05, 0.10, 0.20, 0.30, 0.40, 0.50, 1.00, 2.00 and 5.00 V/s without ferrocene as internal standard. *Bottom:* a wide scan including the ligand reduction peaks at a scan rate of 0.10 V/s. All scans initiated in the positive direction from ca -0.3 V,

**Fig. 11:**
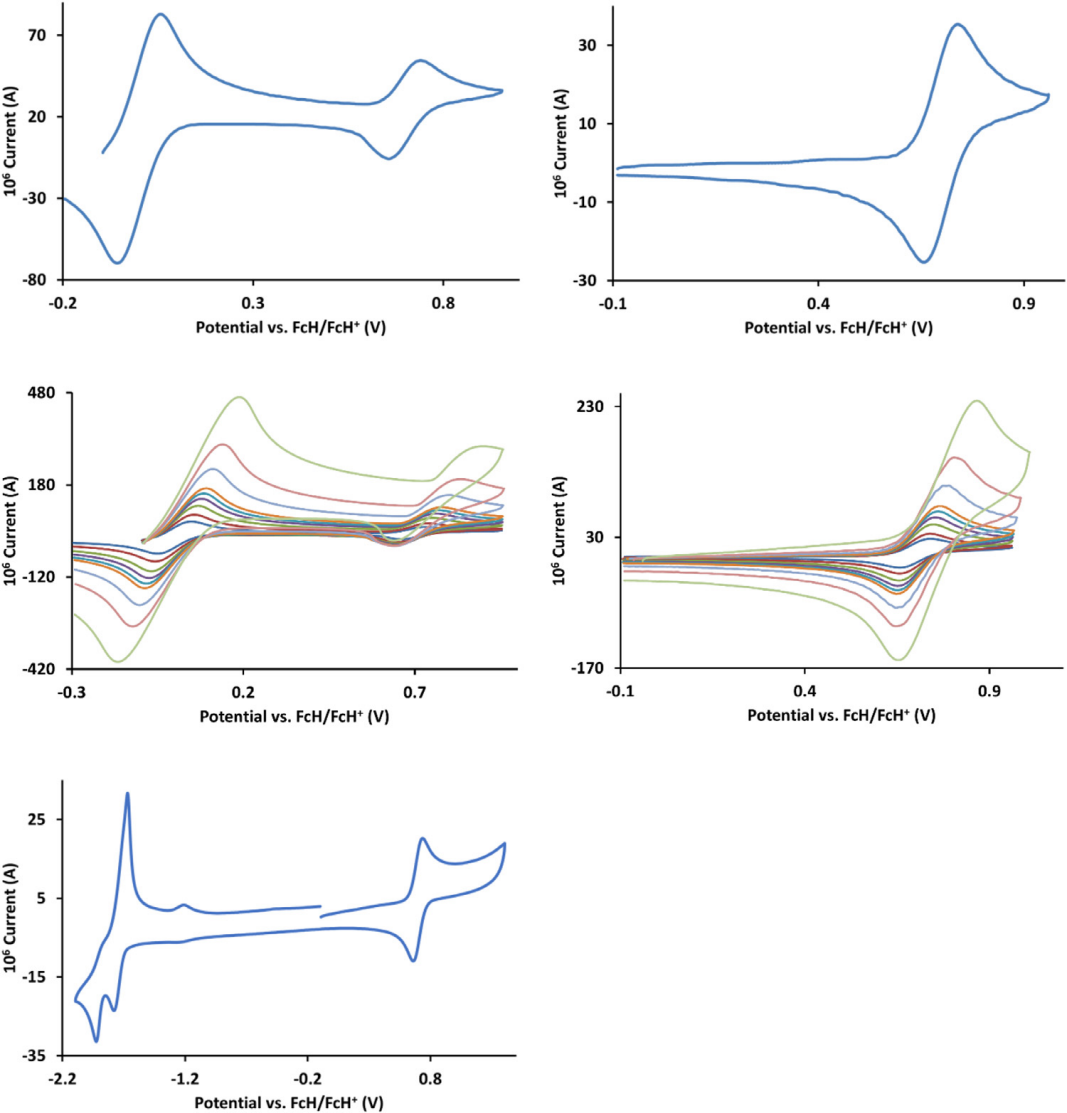
Cyclic voltammograms of *tris*(1,10-phenanthroline)iron(II) perchlorate, [Fe(phen)_3_](ClO_4_)_2_, 9, in CH_3_CN as solvent with [NBu_4_][PF_6_]) as supporting electrolyte. Details are provided in the caption of Fig. 10.

**Fig. 12:**
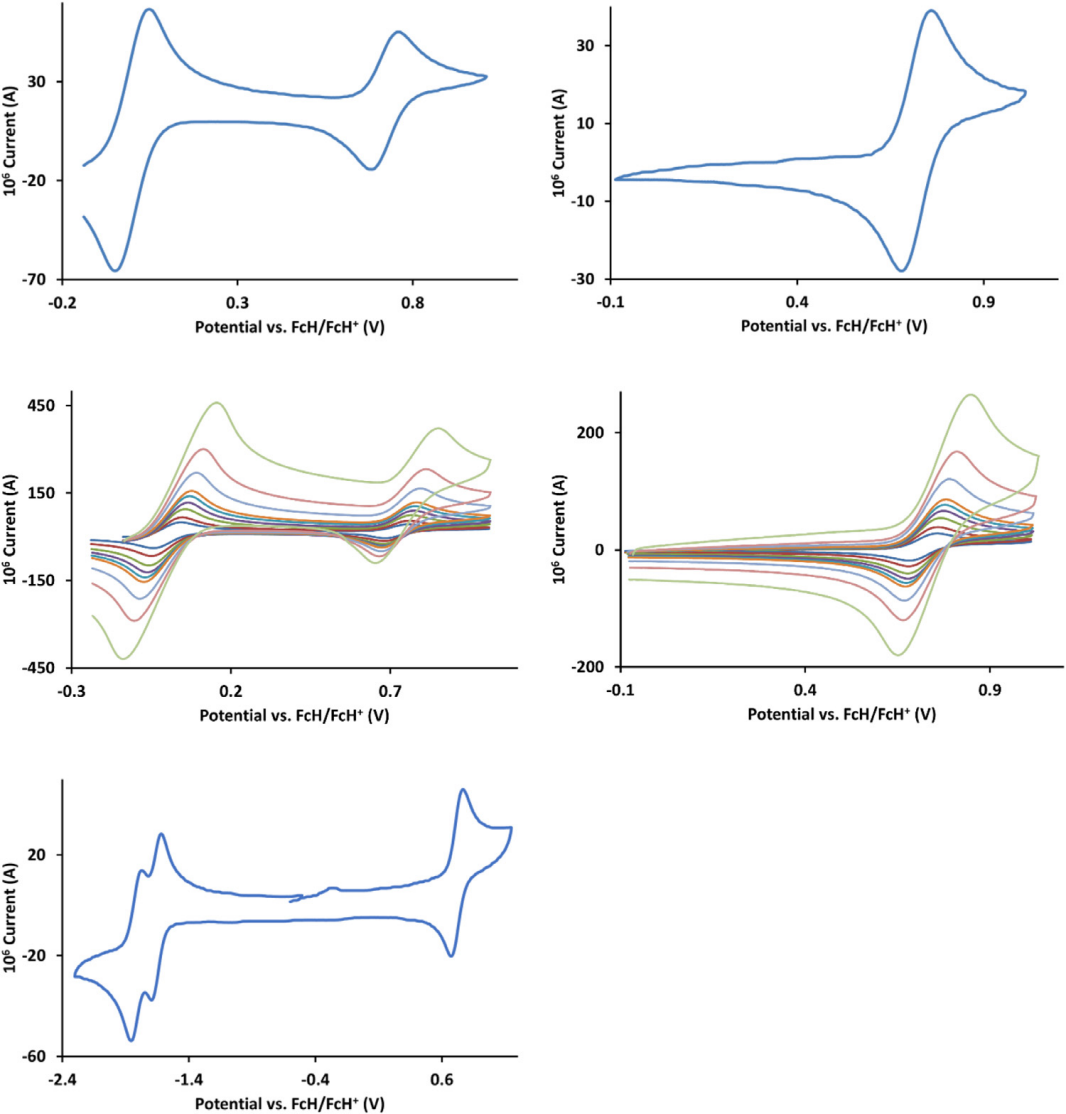
Cyclic voltammograms of *tris*(2,2’:6,2”-terpyridine)iron(II) perchlorate, [Fe(2,2’:6,2”-tpy)_2_](ClO_4_)_2_, 10, in CH_3_CN as solvent with [NBu_4_][PF_6_]) as supporting electrolyte. Details are provided in the caption to Fig. 10.

**Fig. 13:**
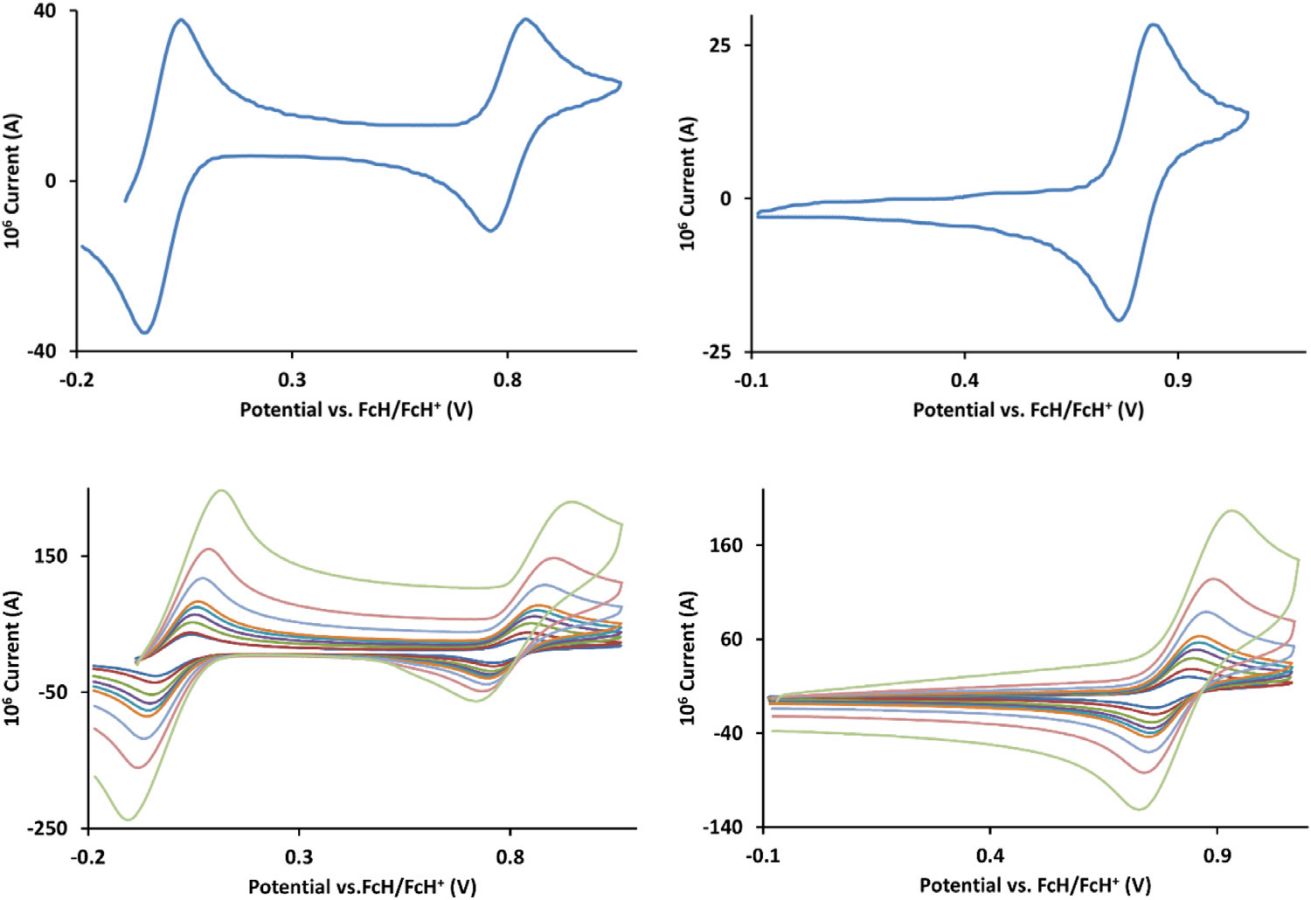
Cyclic voltammograms of *tris*(5,6-epoxy-5,6-dihydro-1,10-phenanthroline)iron(II) perchlorate, [Fe(5,6-epoxy-5,6-OH-phen)_3_](ClO_4_)_2_, 11, in CH_3_CN as solvent with [NBu_4_][PF_6_]) as supporting electrolyte. Details are provided in the caption to Fig. 3.

**Fig. 14:**
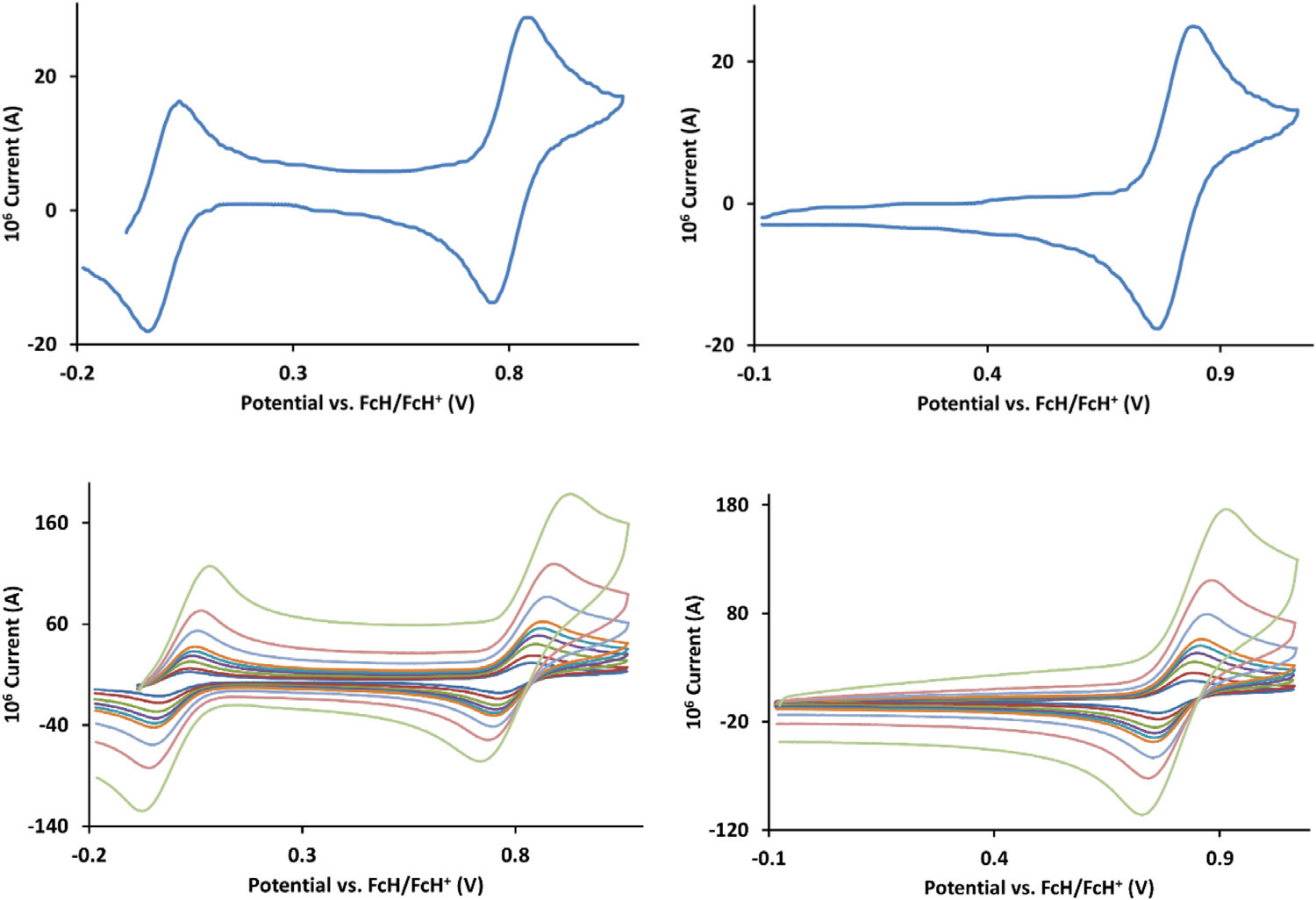
Cyclic voltammograms of *tris*(5-chloro-1,10-phenanthroline)iron(II) perchlorate, [Fe(5-Cl-phen)_3_](ClO_4_)_2_, 12, in CH_3_CN as solvent with [NBu_4_][PF_6_]) as supporting electrolyte. Details are provided in the caption to Fig. 3.

**Fig. 15:**
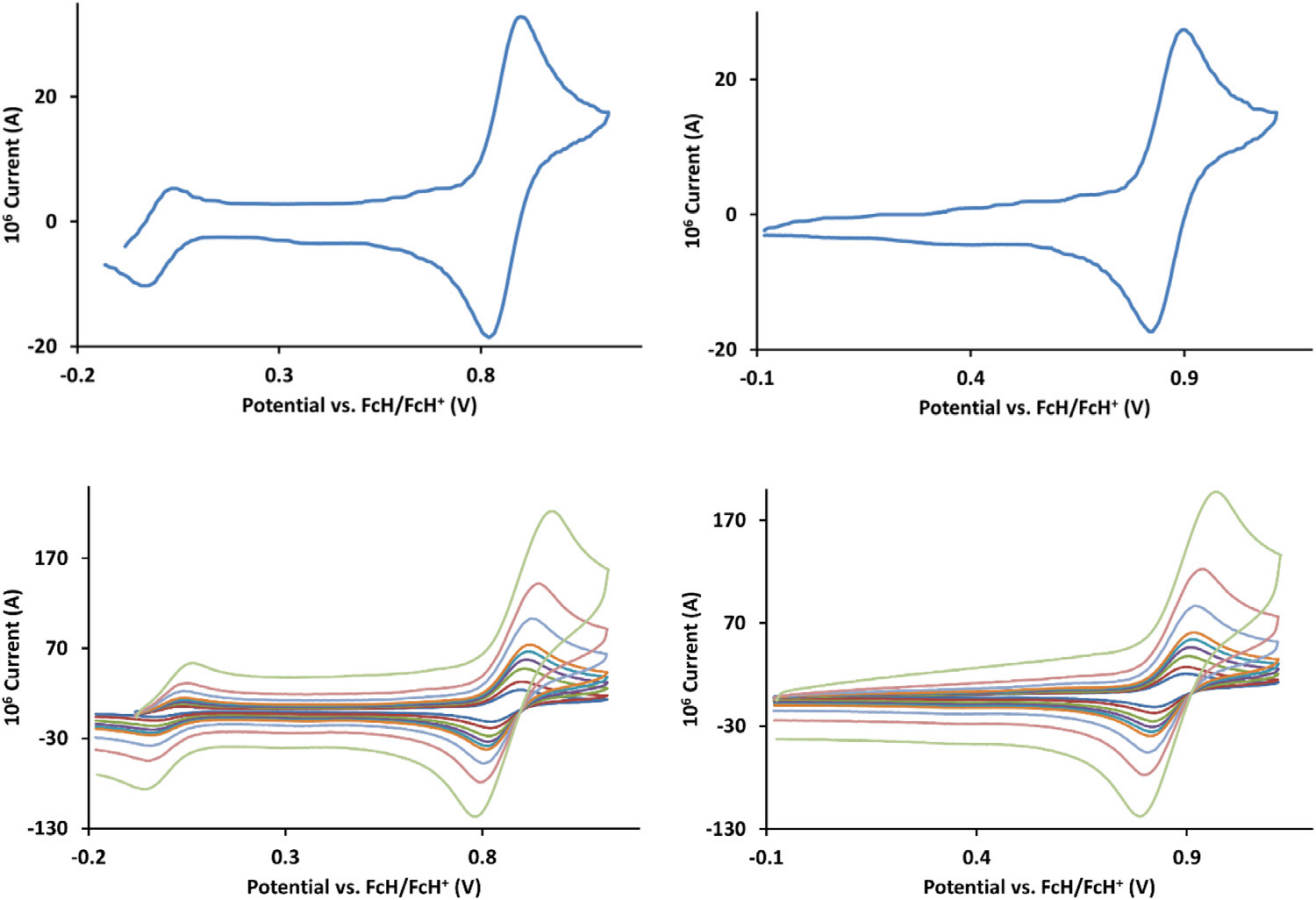
Cyclic voltammograms of *tris*(4,7-dichloro-1,10-phenanthroline)iron(II) perchlorate, [Fe(4,7-Cl-phen)_3_](ClO_4_)_2_, 13, in CH_3_CN as solvent with [NBu_4_][PF_6_]) as supporting electrolyte. Details are provided in the caption to Fig. 3.

**Fig. 16:**
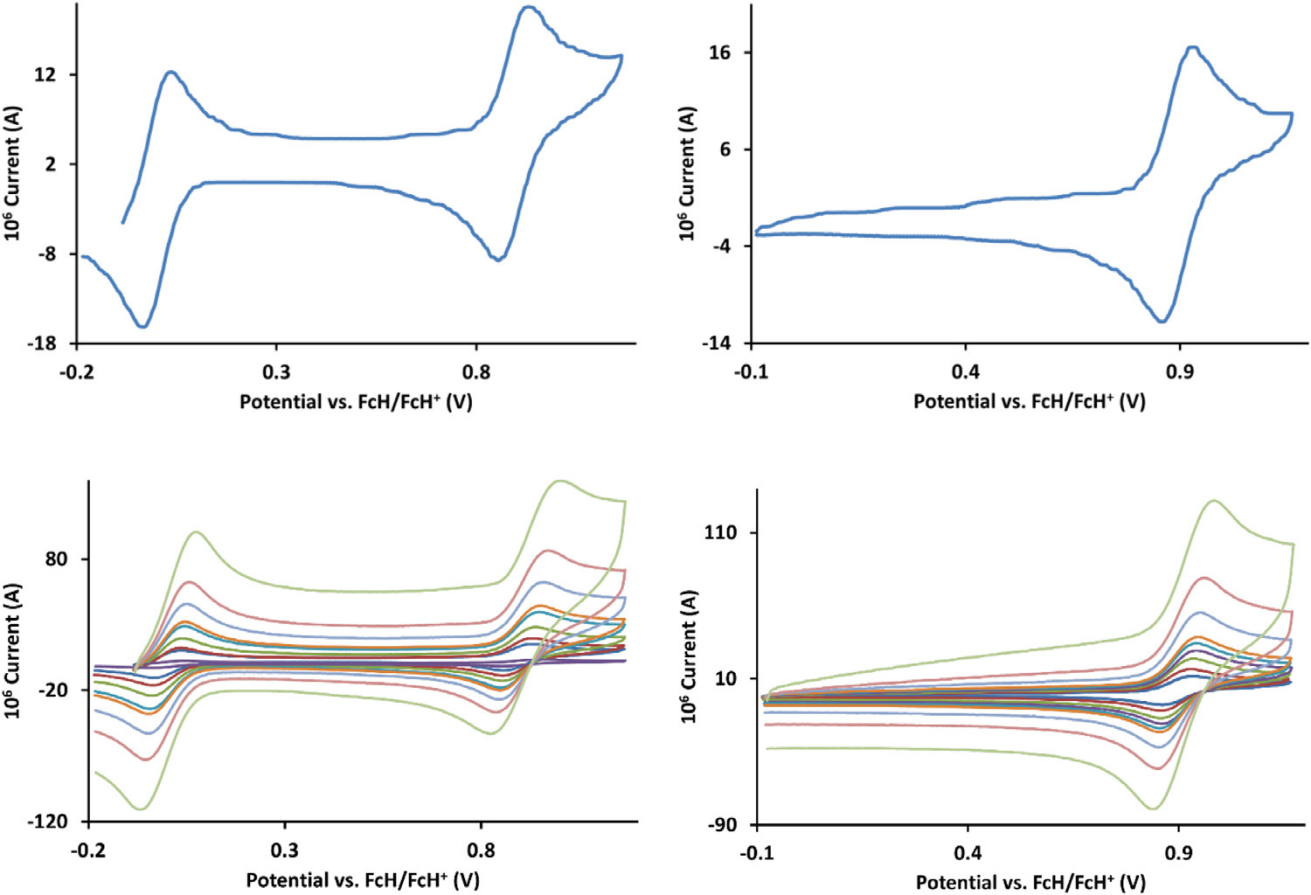
Cyclic voltammograms of *tris*(5-nitro-1,10-phenanthroline)iron(II) perchlorate, [Fe(5-NO_2_-phen)_3_](ClO_4_)_2_, 14, in CH_3_CN as solvent with [NBu_4_][PF_6_]) as supporting electrolyte. Details are provided in the caption to Fig. 3.

The redox data of fourteen octahedral Fe^II^ complexes are presented in this report. These complexes, **1**–**14**, contain 1,10-phenanthroline and substituted 1,10-phenanthroline, bipyridine and substituted bipyridine, and terpyridine ligands, see Fig. 1 for the complex series of this study. The substituents on complexes **1** – **14** vary from electron donating (OMe and Me) to electron withdrawing (Cl and NO_2_). Comparative CVs of **1** – **14**, at a scan rate of 0.10 Vs^−1^ CVs [1] are shown in Fig. 2. The data presented in this article are related to the research article, “Electronic Properties of Fe Charge Transfer Complexes – a Combined Experimental and Theoretical Approach” [1]. The redox data obtained for the Fe^II^ complexes are of practical use in its applications as redox indicators, catalysts and photo-active mediators in, amongst others, dye sensitized solar cells [2], [3], [4]. All electrochemical data were obtained from cyclic voltammograms, at scan rates that varied from 0.05 Vs^−1^ to 5.00 Vs^−1^, See Fig. 3-16, with corresponding data tabulated in Table 1, Table 2, Table 3, Table 4, Table 5, Table 6, Table 7, Table 8, Table 9, Table 10, Table 11, Table 12, Table 13, Table 14 (data for 0.10 Vs^−1^ CVs from [1]).

**Table 1:** *Tris*(4,4’-dimethoxy-2,2’-bipyridine)iron(II) perchlorate, 1, electrochemical data (potential in V vs. FcH/FCH^+^) of the Fe^II/III^ redox couple in acetonitrile(CH_3_CN) with [NBu_4_][PF_6_]) as supporting electrolyte, for *ca* 0.002 mol dm^−3^ complex solution at the indicated scan rates.

Scan Rate (V/s)	E_pa_(V)	10^6^ I_pa_ (A)	E_pc_ (V)	10^6^ I_pc_ (A)	E°’ (V)	ΔE(V)	I_pc_/I_pa_
0.05	0.404	16.23	0.320	16.02	0.362	0.084	0.99
0.10	0.405	24.25	0.319	23.98	0.362	0.086	0.99
0.20	0.410	33.25	0.314	32.85	0.362	0.096	0.99
0.30	0.414	37.32	0.310	36.97	0.362	0.104	0.99
0.40	0.419	52.02	0.305	50.98	0.362	0.114	0.98
0.50	0.423	54.64	0.301	53.98	0.362	0.122	0.99
1.00	0.432	57.43	0.292	56.35	0.362	0.140	0.98
2.00	0.445	78.35	0.279	76.98	0.362	0.166	0.98
5.00	0.474	115.32	0.250	112.98	0.362	0.224	0.98

**Table 2:** *Tris*(3,4,7,8-tetramethyl-1,10-phenanthroline)iron(II) perchlorate, 2, electrochemical data (potential in V vs. FcH/FcH^+^) of the Fe^II/III^ redox couple in acetonitrile(CH_3_CN) with [NBu_4_][PF_6_]) as supporting electrolyte, for *ca* 0.002 mol dm^−3^ complex solution at the indicated scan rates.

Scan Rate (V/s)	E_pa_(V)	10^6^ I_pa_ (A)	E_pc_ (V)	10^6^ I_pc_ (A)	E°’ (V)	ΔE(V)	I_pc_/I_pa_
0.05	0.488	5.03	0.416	4.89	0.452	0.072	0.97
0.10	0.488	5.89	0.416	5.69	0.452	0.072	0.97
0.20	0.490	11.22	0.414	10.84	0.452	0.076	0.97
0.30	0.490	12.35	0.414	11.98	0.452	0.076	0.97
0.40	0.495	14.23	0.409	13.89	0.452	0.086	0.98
0.50	0.500	16.12	0.404	15.79	0.452	0.096	0.98
1.00	0.498	20.54	0.406	19.86	0.452	0.092	0.97
2.00	0.504	22.65	0.400	21.98	0.452	0.104	0.97
5.00	0.518	47.35	0.386	46.45	0.452	0.132	0.98

**Table 3:** *Tris*(4,4’-dimethyl-2,2’-bipyridine)iron(II) perchlorate), 3, electrochemical data (potential in V vs. FcH/FcH^+^) of the Fe^II/III^ redox couple in acetonitrile(CH_3_CN) with [NBu_4_][PF_6_]) as supporting electrolyte, for *ca* 0.002 mol dm^−3^ complex solution at the indicated scan rates.

Scan Rate (V/s)	E_pa_(V)	10^6^ I_pa_ (A)	E_pc_ (V)	10^6^ I_pc_ (A)	E°’ (V)	ΔE(V)	I_pc_/I_pa_
0.05	0.561	18.25	0.486	17.96	0.523	0.075	0.98
0.10	0.562	21.23	0.485	20.87	0.523	0.077	0.98
0.20	0.567	34.62	0.479	33.98	0.523	0.088	0.98
0.30	0.571	51.76	0.475	50.92	0.523	0.096	0.98
0.40	0.574	56.23	0.472	54.98	0.523	0.102	0.98
0.50	0.578	59.89	0.468	58.42	0.523	0.110	0.98
1.00	0.586	78.88	0.461	76.95	0.523	0.125	0.98
2.00	0.595	98.72	0.451	97.15	0.523	0.144	0.98
5.00	0.618	157.45	0.428	155.08	0.523	0.190	0.98

**Table 4:** *Tris*(5-amino-1,10-phenanthroline)iron(II) perchlorate), 4, electrochemical data (potential in V vs. FcH/FcH^+^) of the Fe^II/III^ redox couple in acetonitrile(CH_3_CN) with [NBu_4_][PF_6_]) as supporting electrolyte, for *ca* 0.002 mol dm^−3^ complex solution at the indicated scan rates.

Scan Rate (V/s)	E_pa_(V)	10^6^ I_pa_ (A)	E_pc_ (V)	10^6^ I_pc_ (A)	E°’ (V)	ΔE(V)	I_pc_/I_pa_
0.05	0.620	9.58	0.544	9.39	0.582	0.076	0.98
0.10	0.620	12.26	0.544	11.98	0.582	0.076	0.98
0.20	0.624	20.98	0.540	20.55	0.582	0.084	0.98
0.30	0.629	24.33	0.535	23.87	0.582	0.094	0.98
0.40	0.633	30.63	0.532	29.98	0.582	0.101	0.98
0.50	0.634	32.08	0.530	31.36	0.582	0.104	0.98
1.00	0.640	38.65	0.523	37.98	0.582	0.117	0.98
2.00	0.648	51.76	0.516	50.98	0.582	0.132	0.98
5.00	0.670	83.69	0.494	81.87	0.582	0.176	0.98

**Table 5:** *Tris*(4-methyl-1,10-phenanthroline)iron(II) perchlorate, 5, electrochemical data (potential in V vs. FcH/FcH^+^) of the Fe^II/III^ redox couple in acetonitrile(CH_3_CN) with [NBu_4_][PF_6_]) as supporting electrolyte, for *ca* 0.002 mol dm^−3^ complex solution at the indicated scan rates.

Scan Rate (V/s)	E_pa_(V)	10^6^ I_pa_ (A)	E_pc_ (V)	10^6^ I_pc_ (A)	E°’ (V)	ΔE(V)	I_pc_/I_pa_
0.05	0.646	4.15	0.578	4.03	0.612	0.068	0.97
0.10	0.652	7.13	0.572	6.96	0.612	0.080	0.98
0.20	0.652	7.68	0.573	7.45	0.612	0.079	0.97
0.30	0.656	10.58	0.569	10.21	0.612	0.087	0.97
0.40	0.656	11.35	0.568	10.98	0.612	0.088	0.97
0.50	0.659	13.98	0.565	13.65	0.612	0.094	0.98
1.00	0.662	17.45	0.562	16.98	0.612	0.100	0.97
2.00	0.665	21.59	0.559	20.89	0.612	0.106	0.97
5.00	0.682	33.69	0.543	32.65	0.612	0.139	0.97

**Table 6:** *Tris*(5,6-dimethyl-1,10-phenanthroline)iron(II) perchlorate), 6, electrochemical data (potential in V vs. FcH/FcH^+^) of the Fe^II/III^ redox couple in acetonitrile(CH_3_CN) with [NBu_4_][PF_6_]) as supporting electrolyte, for *ca* 0.002 mol dm^−3^ complex solution at the indicated scan rates.

Scan Rate (V/s)	E_pa_(V)	10^6^ I_pa_ (A)	E_pc_ (V)	10^6^ I_pc_ (A)	E°’ (V)	ΔE(V)	I_pc_/I_pa_
0.05	0.678	9.60	0.603	9.42	0.641	0.075	0.98
0.10	0.677	13.04	0.604	12.68	0.641	0.073	0.97
0.20	0.684	21.35	0.596	20.68	0.640	0.088	0.97
0.30	0.685	24.15	0.595	23.39	0.640	0.090	0.97
0.40	0.692	26.55	0.588	25.98	0.640	0.104	0.98
0.50	0.690	28.35	0.591	27.89	0.641	0.099	0.98
1.00	0.698	39.14	0.582	38.39	0.640	0.116	0.98
2.00	0.706	54.12	0.575	52.68	0.641	0.131	0.97
5.00	0.720	72.98	0.560	71.35	0.640	0.160	0.98

**Table 7:** *Tris*(5-methyl-1,10-phenanthroline)iron(II) perchlorate, 7, electrochemical data (potential in V vs. FcH/FcH^+^) of the Fe^II/III^ redox couple in acetonitrile(CH_3_CN) with [NBu_4_][PF_6_]) as supporting electrolyte, for *ca* 0.002 mol dm^−3^ complex solution at the indicated scan rates.

Scan Rate (V/s)	E_pa_(V)	10^6^ I_pa_ (A)	E_pc_ (V)	10^6^ I_pc_ (A)	E°’ (V)	ΔE(V)	I_pc_/I_pa_
0.05	0.701	8.88	0.637	8.59	0.669	0.064	0.97
0.10	0.704	12.35	0.633	11.98	0.669	0.071	0.97
0.20	0.708	16.68	0.631	16.2	0.669	0.077	0.97
0.30	0.710	18.98	0.627	18.34	0.669	0.083	0.97
0.40	0.713	24.66	0.624	23.88	0.669	0.089	0.97
0.50	0.716	27.35	0.622	26.59	0.669	0.094	0.97
1.00	0.717	35.85	0.621	34.87	0.669	0.096	0.97
2.00	0.726	54.12	0.611	52.68	0.669	0.115	0.97
5.00	0.745	68.97	0.592	67.23	0.669	0.153	0.97

**Table 8:** *Tris*(2,2’-bipyridine)iron(II) perchlorate, 8, electrochemical data (potential in V vs. FcH/FcH^+^) of the Fe^II/III^ redox couple in acetonitrile(CH_3_CN) with [NBu_4_][PF_6_]) as supporting electrolyte, for *ca* 0.002 mol dm^−3^ complex solution at the indicated scan rates.

Scan Rate (V/s)	E_pa_(V)	10^6^ I_pa_ (A)	E_pc_ (V)	10^6^ I_pc_ (A)	E°’ (V)	ΔE(V)	I_pc_/I_pa_
0.05	0.724	18.46	0.639	17.97	0.682	0.085	0.97
0.10	0.729	24.53	0.636	23.86	0.682	0.093	0.97
0.20	0.737	32.64	0.627	31.98	0.682	0.110	0.98
0.30	0.742	37.69	0.623	36.46	0.682	0.119	0.97
0.40	0.747	45.38	0.616	44.17	0.682	0.131	0.97
0.50	0.749	54.42	0.615	53.53	0.682	0.134	0.98
1.00	0.759	66.91	0.604	65.33	0.682	0.155	0.98
2.00	0.771	82.62	0.593	81.12	0.682	0.178	0.98
5.00	0.803	104.92	0.560	103.03	0.682	0.243	0.98

**Table 9:** *Tris*(1,10-phenanthroline)iron(II) perchlorate, 9, electrochemical data (potential in V vs. FcH/FcH^+^) of the Fe^II/III^ redox couple in acetonitrile(CH_3_CN) with [NBu_4_][PF_6_]) as supporting electrolyte, for *ca* 0.002 mol dm^−3^ complex solution at the indicated scan rates.

Scan Rate (V/s)	E_pa_(V)	10^6^ I_pa_ (A)	E_pc_ (V)	10^6^ I_pc_ (A)	E°’ (V)	ΔE(V)	I_pc_/I_pa_
0.05	0.736	21.84	0.659	21.36	0.698	0.077	0.98
0.10	0.739	26.41	0.656	25.97	0.698	0.083	0.98
0.20	0.744	45.05	0.652	44.13	0.698	0.092	0.98
0.30	0.747	49.53	0.649	48.61	0.698	0.098	0.98
0.40	0.753	45.38	0.644	44.17	0.698	0.109	0.97
0.50	0.755	54.42	0.640	53.53	0.698	0.115	0.98
1.00	0.756	78.04	0.641	77.63	0.698	0.115	0.99
2.00	0.770	107.06	0.625	106.34	0.698	0.145	0.99
5.00	0.798	163.86	0.597	162.18	0.698	0.201	0.99

**Table 10:** *Tris*(2,2’:6,2”-terpyridine)iron(II) perchlorate, 10, electrochemical data (potential in V vs. FcH/FcH^+^) of the Fe^II/III^ redox couple in acetonitrile(CH_3_CN) with [NBu_4_][PF_6_]) as supporting electrolyte, for *ca* 0.002 mol dm^−3^ complex solution at the indicated scan rates.

Scan Rate (V/s)	E_pa_(V)	10^6^ I_pa_ (A)	E_pc_ (V)	10^6^ I_pc_ (A)	E°’ (V)	ΔE(V)	I_pc_/I_pa_
0.05	0.755	22.38	0.685	21.83	0.720	0.070	0.98
0.10	0.759	28.55	0.682	27.86	0.720	0.077	0.98
0.20	0.762	42.64	0.678	41.69	0.720	0.084	0.98
0.30	0.770	53.94	0.670	52.97	0.720	0.100	0.98
0.40	0.771	67.91	0.669	66.75	0.720	0.102	0.98
0.50	0.774	73.25	0.665	72.13	0.720	0.109	0.98
1.00	0.776	110.01	0.663	106.97	0.720	0.113	0.97
2.00	0.796	135.27	0.644	130.93	0.720	0.152	0.97
5.00	0.813	198.67	0.628	192.38	0.720	0.185	0.97

**Table 11:** *Tris*(5,6-epoxy-5,6-dihydro-1,10-phenanthroline)iron(II) perchlorate, 11, electrochemical data (potential in V vs. FcH/FcH^+^) of the Fe^II/III^ redox couple in acetonitrile(CH_3_CN) with [NBu_4_][PF_6_]) as supporting electrolyte, for *ca* 0.002 mol dm^−3^ complex solution at the indicated scan rates.

Scan Rate (V/s)	E_pa_(V)	10^6^ I_pa_ (A)	E_pc_ (V)	10^6^ I_pc_ (A)	E°’ (V)	ΔE(V)	I_pc_/I_pa_
0.05	0.836	16.21	0.768	15.73	0.802	0.068	0.97
0.10	0.843	20.49	0.762	19.79	0.802	0.081	0.97
0.20	0.844	31.14	0.759	30.43	0.802	0.085	0.98
0.30	0.851	38.67	0.754	37.82	0.802	0.097	0.98
0.40	0.856	43.65	0.748	42.61	0.802	0.108	0.98
0.50	0.860	55.32	0.745	54.35	0.802	0.115	0.98
1.00	0.864	73.05	0.739	70.94	0.802	0.125	0.97
2.00	0.872	94.75	0.732	92.37	0.802	0.140	0.97
5.00	0.905	125.67	0.700	121.51	0.802	0.205	0.97

**Table 12:** *Tris*(5-chloro-1,10-phenanthroline)iron(II) perchlorate, 12, electrochemical data (potential in V vs. FcH/FcH^+^) of the Fe^II/III^ redox couple in acetonitrile(CH_3_CN) with [NBu_4_][PF_6_]) as supporting electrolyte, for *ca* 0.002 mol dm^−3^ complex solution at the indicated scan rates.

Scan Rate (V/s)	E_pa_(V)	10^6^ I_pa_ (A)	E_pc_ (V)	10^6^ I_pc_ (A)	E°’ (V)	ΔE(V)	I_pc_/I_pa_
0.05	0.840	14.17	0.765	13.84	0.802	0.075	0.98
0.10	0.842	18.36	0.762	17.73	0.802	0.080	0.97
0.20	0.846	29.31	0.757	28.86	0.802	0.089	0.98
0.30	0.849	35.19	0.755	34.64	0.802	0.094	0.98
0.40	0.854	38.97	0.749	37.61	0.802	0.105	0.97
0.50	0.858	42.74	0.747	41.36	0.802	0.111	0.97
1.00	0.861	61.68	0.742	60.53	0.802	0.119	0.98
2.00	0.870	73.96	0.734	72.68	0.802	0.136	0.98
5.00	0.890	110.14	0.714	107.59	0.802	0.176	0.98

**Table 13:** *Tris*(4,7-dichloro-1,10-phenanthroline)iron(II) perchlorate, 13, electrochemical data (potential in V vs. FcH/FcH^+^) of the Fe^II/III^ redox couple in acetonitrile(CH_3_CN) with [NBu_4_][PF_6_]) as supporting electrolyte, for *ca* 0.002 mol dm^−3^ complex solution at the indicated scan rates.

Scan Rate (V/s)	E_pa_(V)	10^6^ I_pa_ (A)	E_pc_ (V)	10^6^ I_pc_ (A)	E°’ (V)	ΔE(V)	I_pc_/I_pa_
0.05	0.895	17.04	0.826	16.57	0.860	0.069	0.97
0.10	0.897	19.61	0.823	18.95	0.860	0.074	0.97
0.20	0.902	28.47	0.819	27.59	0.860	0.083	0.97
0.30	0.906	33.38	0.813	32.87	0.860	0.093	0.98
0.40	0.910	38.39	0.811	37.63	0.860	0.099	0.98
0.50	0.915	43.56	0.805	42.23	0.860	0.110	0.97
1.00	0.917	67.13	0.802	65.91	0.860	0.115	0.98
2.00	0.930	82.11	0.790	79.34	0.860	0.140	0.97
5.00	0.948	122.09	0.772	118.67	0.860	0.176	0.97

**Table 14:** *Tris*(5-nitro-1,10-phenanthroline)iron(II) perchlorate, 14, electrochemical data (potential in V vs. FcH/FcH^+^) of the Fe^II/III^ redox couple in acetonitrile(CH_3_CN) with [NBu_4_][PF_6_]) as supporting electrolyte, for *ca* 0.002 mol dm^−3^ complex solution at the indicated scan rates.

Scan Rate (V/s)	E_pa_(V)	10^6^ I_pa_ (A)	E_pc_ (V)	10^6^ I_pc_ (A)	E°’ (V)	ΔE(V)	I_pc_/I_pa_
0.05	0.925	11.25	0.860	10.94	0.893	0.065	0.97
0.10	0.923	13.24	0.863	12.94	0.893	0.060	0.98
0.20	0.933	20.07	0.854	19.37	0.893	0.079	0.97
0.30	0.937	22.34	0.848	21.79	0.893	0.089	0.98
0.40	0.939	26.38	0.847	25.96	0.893	0.092	0.98
0.50	0.941	28.49	0.846	27.62	0.893	0.095	0.97
1.00	0.945	42.68	0.841	41.93	0.893	0.104	0.98
2.00	0.950	54.12	0.836	53.28	0.893	0.114	0.98
5.00	0.957	83.14	0.829	81.36	0.893	0.128	0.98

## 2 Experimental Design, Materials and Methods

Electrochemical studies *via* cyclic voltammetric measurements were done as described previously [1,5,6]. A BAS100B Electrochemical Analyzer linked to a personal computer, controlled by the BAS100W Version 2.3 Software was used. Measurements were done at 293 K under a blanket of purified Argon. Consecutive experiments under similar experimental conditions illustrated all formal oxidation potentials duplicable within 0.005 V. Cyclic voltammetric measurements were performed on 0.002 mol dm^−3^ (or saturated) solutions of complex. Dissolution was complete, in CH_3_CN, containing 0.100 mol dm^−3^ tetrabutylammonium hexafluorophosphate (TBAPF_6_, [NBu_4_][PF_6_]) as supporting electrolyte. A three-electrode cell was used, consisting of a glassy carbon (surface area 3.14 × 10^−6^ m^2^) working electrode, a Pt auxiliary electrode and a Ag/Ag^+^ (0.010 mol dm^−3^ AgNO_3_ in CH_3_CN) reference electrode [7], mounted on a Luggin capillary [8]. The glassy carbon working electrode was polished on a Buhler polishing mat; first with 1 micron and lastly with ¼ micron diamond paste. Scan rates were varied from 0.05 to 5.00 V.s^−1^. All experimental potentials were referenced against the redox couple of ferrocene, FcH/FcH^+^ (IUPAC), as internal standard [9]. *E*_pa_ (*E*_pc_) = anodic (cathodic) peak potential and *i_pa_* (*i_pc_*) = anodic (cathodic) peak current. The peak potential separation is determined by Δ*E*_p_ = *E*_pa_ – *E*_pc_ and peak current ratio by *i_pc_/i_pa_*.

## Declaration of Competing Interest

The authors declare that there is no conflict of interest regarding the publication of this article.

## Appendix Supplementary materials

Image, application 1

**Supporting Information:** Supporting Information file 1 contains cyclic voltammogram wide scans and associated raw data of iron bipyridyl, iron terpyridyl and iron 1,10-phenanthroline. Supporting Information file 2 contains cyclic voltammogram raw data obtained at scan rates **0.05, 0.10, 0.20, 0.30, 0.40, 0.50, 1.00, 2.00 and 5.00 V/s** for all iron polypyridyl complex derivatives here reported. Image, application 2

Image, application 3
